# Long-Lived Species of Bivalves Exhibit Low MT-DNA Substitution Rates

**DOI:** 10.3389/fmolb.2021.626042

**Published:** 2021-03-15

**Authors:** Mathieu Mortz, Aurore Levivier, Nicolas Lartillot, France Dufresne, Pierre U. Blier

**Affiliations:** ^1^ Institut Des Sciences De La Mer De Rimouski, Université Du Québec à Rimouski, Rimouski, QC, Canada; ^2^ Laboratoire De Biométrie et Biologie Evolutive, UMR CNRS, Université Lyon 1, Villeurbanne, France; ^3^ Laboratoire D’écologie Moléculaire, Département De Biologie, Université Du Québec à Rimouski, Rimouski, QC, Canada; ^4^ Laboratoire De Physiologie Intégrative Et Evolutive, Département De Biologie, Université Du Québec à Rimouski, Rimouski, QC, Canada

**Keywords:** life-history evolution, longevity, mitochondrial genome, synonymous substitution rates, Markov chain Monte Carlo, bayesian statistics, bivalve

## Abstract

Bivalves represent valuable taxonomic group for aging studies given their wide variation in longevity (from 1–2 to >500 years). It is well known that aging is associated to the maintenance of Reactive Oxygen Species homeostasis and that mitochondria phenotype and genotype dysfunctions accumulation is a hallmark of these processes. Previous studies have shown that mitochondrial DNA mutation rates are linked to lifespan in vertebrate species, but no study has explored this in invertebrates. To this end, we performed a Bayesian Phylogenetic Covariance model of evolution analysis using 12 mitochondrial protein-coding genes of 76 bivalve species. Three life history traits (maximum longevity, generation time and mean temperature tolerance) were tested against 1) synonymous substitution rates (dS), 2) conservative amino acid replacement rates (Kc) and 3) ratios of radical over conservative amino acid replacement rates (Kr/Kc). Our results confirm the already known correlation between longevity and generation time and show, for the first time in an invertebrate class, a significant negative correlation between dS and longevity. This correlation was not as strong when generation time and mean temperature tolerance variations were also considered in our model (marginal correlation), suggesting a confounding effect of these traits on the relationship between longevity and mtDNA substitution rate. By confirming the negative correlation between dS and longevity previously documented in birds and mammals, our results provide support for a general pattern in substitution rates.

## Introduction

Contemporary evolutionary theories of aging suggest that aging processes are influenced by forces of natural selection that optimize fitness early in life (Hugues and Reynolds, 2005). In this context, uncovering mechanisms which might explain connection between life traits and longevity becomes a major concern, and could provide a better understanding of aging and the pattern of life-history strategies evolution. Since extrinsic mortality is usually high in wild populations, only a small proportion of individuals will survive long enough to experience senescence, hence the strength of natural selection should decline with age. Among the classical evolutionary theories of aging, the “mutation accumulation theory” proposes that late-acting mutations can accumulate, leading to deleterious physiological conditions and aging process (Medawar, 1952). The corollary is that early-acting mutations should be selected against and species period of maturation may depend on the capacity to develop strategies to avoid mutations during the period of accumulation. The “antagonist theory of aging” posits that positive selection of genes that are beneficial early in life can be harmful later in life inducing the evolution of senescence (Williams, 1957). This theory is a refinement of the previous one and proceeds with the same rational: the mutations which operate early in life should be selected (either positive or negative), contrary to the mutations which occur after the reproduction period. The third theory, “the disposable soma” theory, argues that early maturation and reproduction restrict energy available to maintenance and anti-aging mechanisms, resulting in accelerated aging process (Kirkwood, 1977; Kirkwood and Holliday, 1979). These theories suggest evolutionary trade-off between reproduction and lifespan and predict that low mutation rates may facilitate late reproduction in order to reduce mutation accumulations prior to reproduction.

The cellular mechanisms involved in aging processes remain to be fully understood but the relationship between the overall energy expenditure of an organism and its lifespan has been established for decades. The free radical theory of aging described by Harman (Harman, 1956) correlates aging with the accumulation of Reactive Oxygen Species (ROS). These molecules react and affect the function of essential biomolecules (proteins, lipids, and nucleotides) to cause significant dysfunction which accelerated cell senescence. Since mitochondria are the principal sources of ROS, they are also suspected to be the primary target of oxidative stress (Hulbert et al., 2007; Pamplona and Barja, 2011; Munro and Blier, 2012; Munro et al., 2013) and to relate to the rate of aging (Barja, 2004). Mitochondria play major roles in bioenergetics (Nicholls and Ferguson, 2013), autophagy and apoptosis (Ren et al., 2014; Li et al., 2015), and inflammation (Rath and Haller, 2012; Currais, 2015; Thoudam et al., 2016), hence mutation accumulations and functional impairments of mitochondrial protein-coding genes (mtPCGs) should result in failures in the ability to fine-tune homeostasis and therefore leads to senescence. Thus, accumulation of mitochondrial DNA (mtDNA) mutations has been already linked to individual lifespan in vertebrates (Feng et al., 2001; Trifunovic et al., 2004; Yang et al., 2013). If the ROS impacts on mitochondrial structures and genetic material are associated with divergences in species-specific rates of aging, we postulate that long-lived species should have evolved mechanisms to either attenuate oxidative stress effect or to repair dysfunctional molecules.

In this context, comparative studies including short-lived and long-lived species represent a powerful approach to delineate physiological or biochemical traits associated to aging process. In particular, one phylogenetic comparative method (Lartillot and Poujol, 2011) has been successfully employed to investigate the evolutionary variations of longevity and substitution rates in fish, mammals and birds (Galtier et al., 2009a; Galtier et al., 2009b; Hua et al., 2015). As expected, in these previous studies, longevity is negatively correlated with nuclear DNA and mtDNA mutation rates, and dN/dS (the ratio of non-synonymous to synonymous substitution rates), and Kr/Kc (the ratio of radical to conservative amino-acids substitutions) showed a positive correlation both in nuclear and mt-DNA. It is not yet possible to conclude if this relationship reflects a primordial rule of aging process or if it is specific to vertebrates.

In order to solve this problem, we decided to scrutinize the patterns of mtDNA substitutions in invertebrates. Bivalves have been advocated as exceptional models to study longevity (Blier et al., 2017) because 1) they have an exceptional range of lifespan (Guo, 2009; Butler et al., 2012); 2) measuring their age is easy as each year they deposit a growth ring in the inner face of their shell (Richardson, 2001) and, 3) they range from the poles to the tropics and are therefore adapted to a wide variety of habitats (Philipp et al., 2005; Silva Cavalcanti and Costa 2011). Bivalves have a wider range of lifespan than mammals or birds, which are currently used as longevity models in comparative studies [maximum recorded longevity inferior to 250 years] (Tacutu et al., 2013). Recently, a 507 years old bivalve *Arctica islandica* has been found in Iceland coasts, making it the longest-lived non-colonial animal (Butler et al., 2012). Few other bivalves can live more than 150 years: *Margaritifera margaritifera* (210 years) (Ziuganov et al., 2000) and *Panopea abrupta* (163 years) (Bureau, 2002). In contrast, some bivalve species live only one or two years, such as *Argopecten irradians* and *Musculista senhousia* (Mistri, 2002; Guo, 2009). Moreover, age at sexual maturity is positively and strongly correlated with longevity (Ridgway et al., 2011; Abele and Philipp, 2013). For example, *Margaritifera margaritifera* which can live over 210 years reaches sexual maturity at 20 years (Bauer, 1987) whereas *Musculista senhousia* that lives only two years reaches sexual maturity before one year old (Mistri, 2002). Bivalves with longest lifespan live generally in cold waters whereas those with shortest longevity live in the tropics (Cardoso and Veloso, 2003; Philipp et al., 2005), but we can observe important ranges of maximum lifespan in both habitats.

Lartillot and Pujol (2011) introduced a Bayesian phylogenetic reconstruction and Markov chain Monte Carlo (MCMC) method to correlate and associate the evolution of molecular and phenotypic characters. This method jointly estimates divergence times, substitution rates, and their correlations with life history or any phenotypic traits (Lartillot and Pujol, 2011) and has already been applied to mammal and bird data (Galtier et al., 2009a; Galtier et al., 2009b; Lartillot and Pujol, 2011; Nabholz et al., 2011; Nabholz et al., 2013; Hua et al., 2015). Several substitution parameters are commonly measured: the synonymous substitution rate (dS), the ratio of non-synonymous over synonymous substitution rates (dN/dS) and the ratio of radical over conservative amino-acid replacement rates (Kr/Kc) (Nabholz et al., 2008; Nabholz et al., 2013). Considering the putative role of the mitochondrial encoded proteins in the management of ROS production as well as their thermal-sensitivity (Blier et al., 2014) we suspect that signature of elongated lifespan or any associated life history trait will lead a specific signature in bivalve mitochondrial genomes. Therefore, it will be of a particular interest to study the correlation between mutation rates of mtDNA and life-history traits like longevity in bivalves. It is important to note that some bivalves have a biparental mtDNA transmission, named doubly uniparental inheritance (DUI), different from the strictly maternal inheritance of mtDNA which largely predominates in the animal kingdom (Zouros, 2013). As a consequence, DUI bivalves show the two distinct mtDNA: the F (for “Female inherited”) genome, which participates to the energy production in all somatic tissues, and the M (for “Male inherited”) genome found in gonads and which only contributes to the sperm function (Zouros, 2013; Dégletagne et al., 2016). In this study, we only considered the F genome for DUI species, since it is the principal genome involved in the metabolic activity of somatic tissues.

We present here the first relationship between three phenotypic traits (longevity, generation time and mean temperature tolerance) and mtDNA evolution in invertebrates based on 76 bivalve species. Our confirmation of a negative correlation between the rate of neutral substitution in mitochondrial DNA and longevity strongly indicate that the relationship might be a general rule as it is also found in vertebrates. Further bivalve studies are warranted to delineate precise mechanisms of aging.

## Results and Discussion

### Bivalves Phylogeny and Evolution of mtPCGs Among Lineages

**FIGURE 1:**
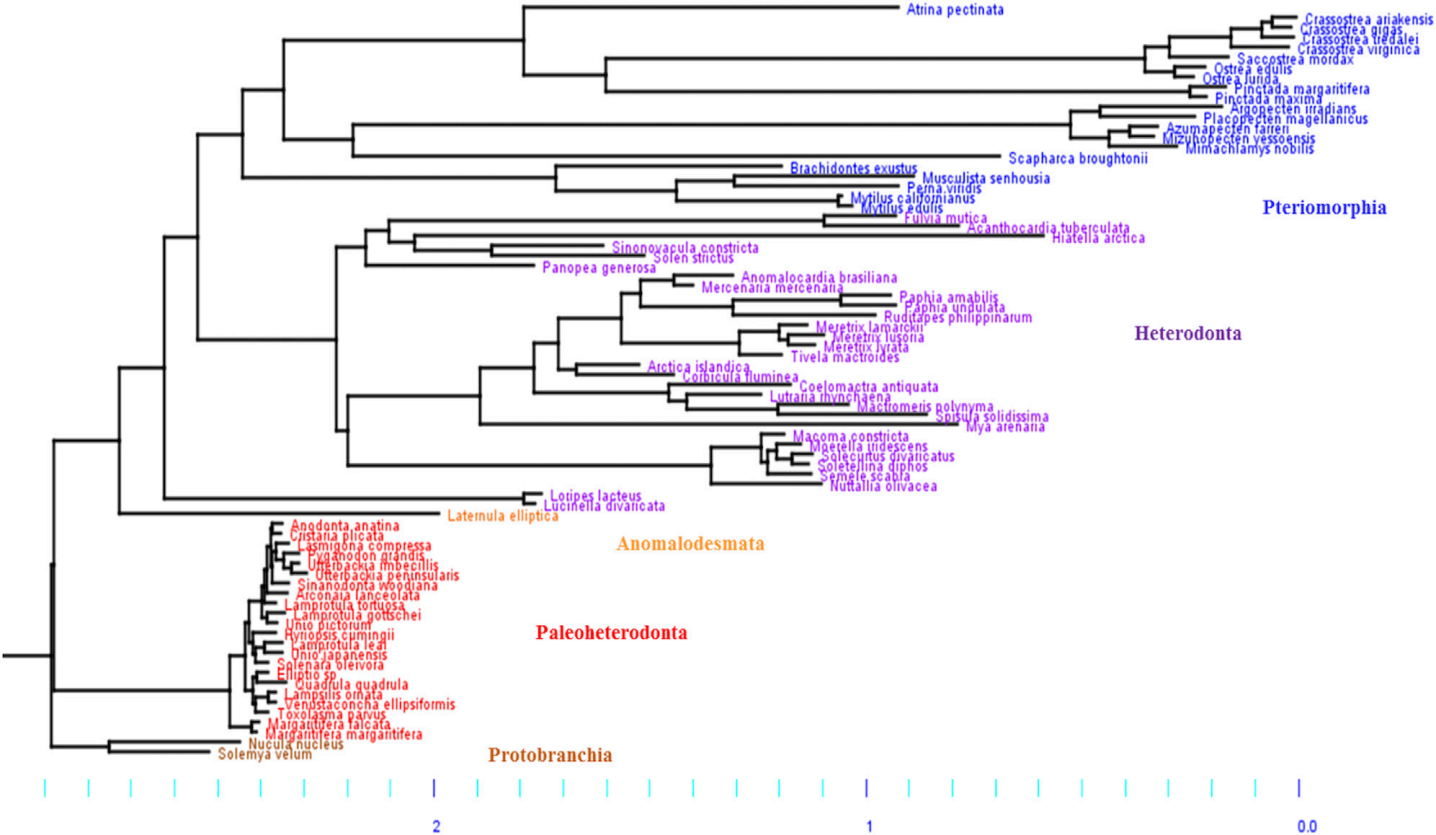
Phylogenetic Bayesian tree of mitochondrial DNA in bivalves, obtained by using the site-heterogeneous CAT-GTR model. The five subclasses of bivalves were retrieved: Pteriomorphia (blue), Heterodonta (purple), Anomalodesmata (orange), Palaeoheterodonta (red) and Protobranchia (brown, used here as outgroup). The branch lengths highlight differences of mitochondrial mutation rate between bivalve subgroups, with two main groups: 1) Paleoheterodonta and Protobranchia, and 2) Anomalodesmata, Heterodonta and Pteriomorphia, which evolved slowler and faster, respectively.

Based on our phylogeny, the five subclasses of Bivalves were retrieved: Protobranchia, Paleoheterodonta, Heterodonta, Pteromorphia and Anomalodesmata (Figure 1), which can be regrouped in three main groups. The first group includes two Protobranchia species (*Nucula nucleus* and *Solemya velum*) and is used as outgroup. The second group includes the Paleoheterodonta species (posterior probability, *pp* = 1) and the third group includes the Heterodonta, Pteriomorphia and Anomalodesmata species (*pp* = 1). The only species in the Anomalodesmata sub-order (*Laternulla elliptica*) is genetically similar to the two Imparidentia species *L. divarticata* and *L. lacteus* (*pp* = 0.95). Numerous studies, to date, have attempted to reconstruct the phylogeny of bivalves based on morphological and/or DNA (mt and/or nuclear) characters (Cope, 1996; Adamkewick et al., 1997; Giribet and Wheeler, 2002; Dreyer et al., 2003; Doucet-Beaupré et al., 2010; Plazzi and Passamonti, 2010; Plazzi et al., 2011; Bieler et al., 2014). In our phylogeny, we found that the Paleoheterodonta was the most basal subclass of the Autrobranchia, but with null support. Conversely, the most recent phylogeny of Bivalvia (Bieler et al., 2014; González et al., 2015) placed the Pteriomorphia as the most basal group of Autobranchia. The places of the other four subclasses are not well resolved yet.

**FIGURE 2:**
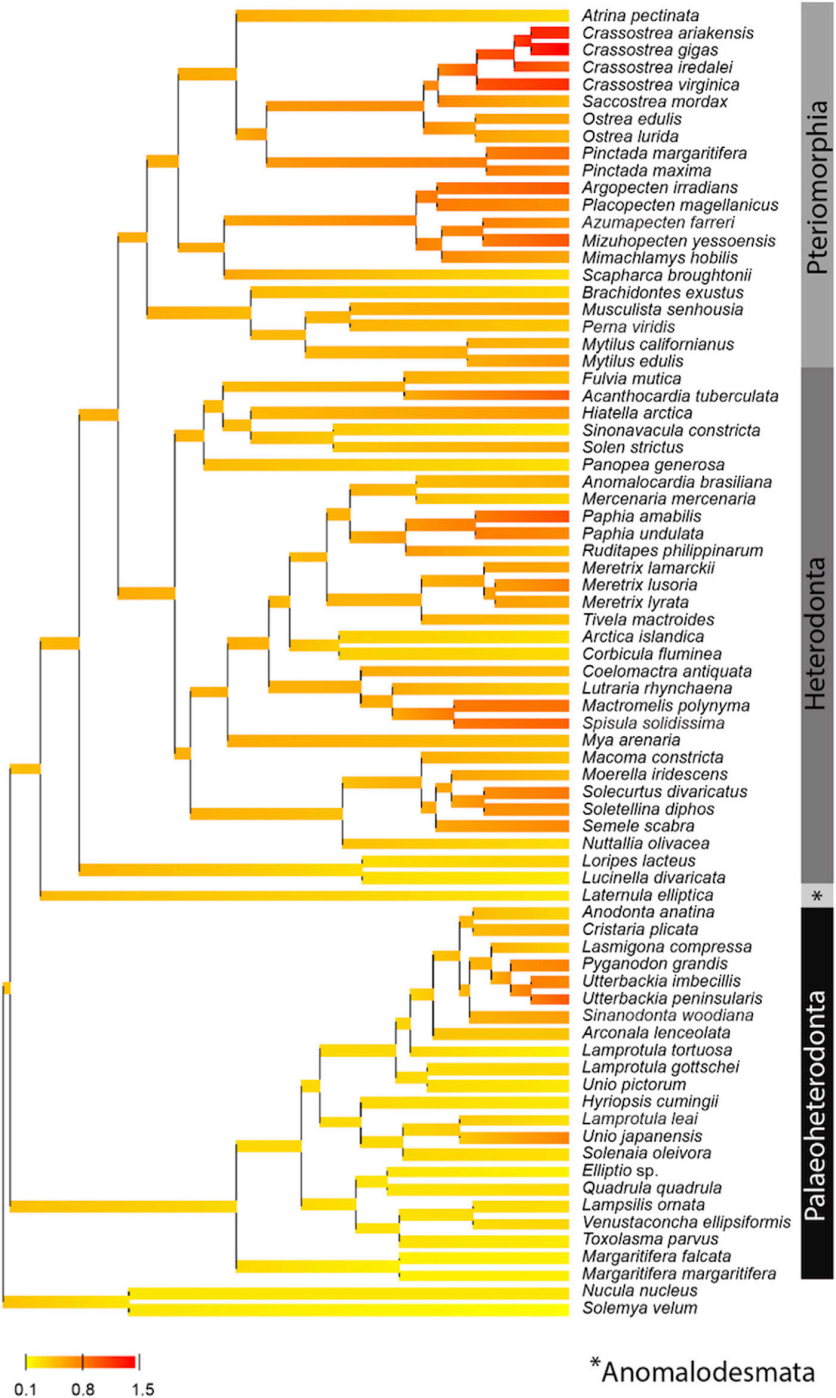
Posterior mean reconstruction of the evolution of dS along phylogeny of mtDNA in bivalves. Branch lengths are proportional to time, and the colors yellow and red correspond to low (0.8–0.1) and high (0.8–1.5) dS, respectively. This representation highlights a high rate of dS (>1) for the genus *Crassostrea* (Pteriomorphia) and a low synonymous substitution rate (<0.5) for protobranchs and most of the Paleoheterodonta species.

Based on the phylogenetic Bayesian tree branch lengths observation (Figure 1), two groups of bivalves emerged: 1) the Palaeoheterodonta, and 2) the Anomalodesmata, Heterodonta and Pteriomorphia, which evolved slower and faster respectively. This was easily confirmed by measuring the overall mean p-distance for each studied subgroup, with a 2-fold less value for Palaeoheterodonta (20.3% ± 0.002) species than those measured for Heterodonta (37.6% ± 0.003) and Pteriomorphia (42.8% ± 0.003) species. In the same way, a similar trend is observed when regarding the evolution of the synonymous substitution rate among lineages (Figure 2). In particular, the genus *Crassostrea* (Pteriomorphia) has the highest rate of dS (>1). The protobranchs and most of the Paleoheterodonta have the lowest synonymous substitution rate (<0.5). This difference might be explained by the relationship among the substitution rates, mitochondrial genome structure and genome rearrangement. Indeed, Palaeoheterodonta species have their genes on two strands of the mitochondrial genome and have a conserved gene order whereas the others subclasses (Anomalodesmata, Heterodonta and Pteriomorphia) possess all genes on one strand and many rearrangements (Breton et al., 2006; Smith and Snyder, 2007; Doucet-Beaupré et al., 2010). The substitution rate could increase as a result of the increase in the occurrences of genome rearrangement (Xu et al., 2006), or gene order changes could result from an increase in mutation rate. The former hypothesis is associated with the potential disequilibrium in base composition due to a displacement of a gene to another location in the mtDNA. Thus if a gene moves to a new location on the mtDNA, the base frequencies within this gene can be out of equilibrium with the mutational processes typical for its new position and this will lead to a rapid burst of substitutions until equilibrium in the base frequencies is reached. The alternative hypothesis i.e., where the gene order rearrangement rate increases as a result of the increase in substitution rate might occur if this increase leads to the creation of repeated/similarities sequences that are prone to recombination (Xu et al., 2006). The high mutation rate found in the present study and the high rate of gene rearrangements (Gissi et al., 2008) for Anomalodesmata, Heterodonta and Pteriomorphia subclasses support this scenario suggesting a strong relationship between the rate of molecular evolution and genome rearrangements in bivalve mitochondrial genomes.

**FIGURE 3:**
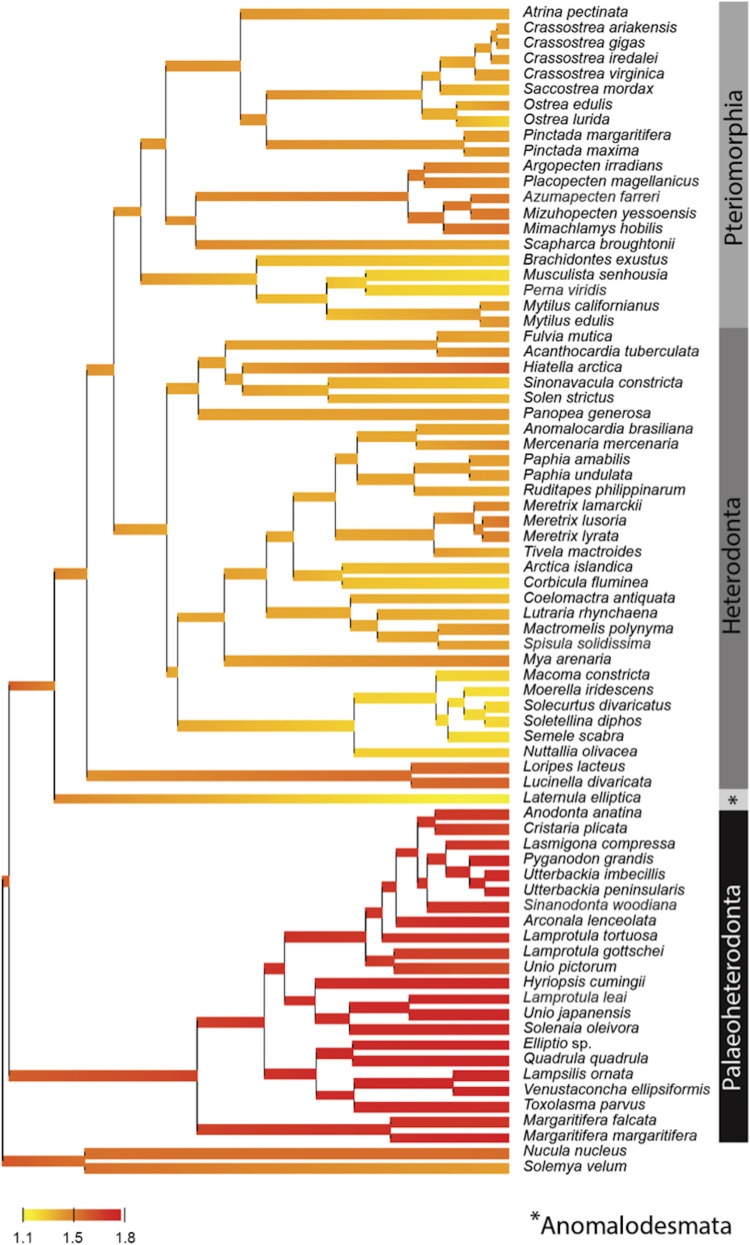
Posterior mean reconstruction of the evolution of radical over conservative amino-acid replacement rates (Kr/Kc) along phylogeny of mtDNA in bivalves. Branch lengths are proportional to time, and the colors yellow and red correspond to low (1.1–1.5) and high (1.5–1.8) Kr/Kc, respectively. This representation highlights a high Kr/Kc (>1.5) for Palaeoheterodonta species, suggesting a specific positive selection pressure for this subclass of bivalves.

By contrast, the evolution of the mtPCGs at the amino-acids level reflected by the evolution of the ratio of radical over conservative amino-acid replacement rates (Kr/Kc) among bivalve lineages (Figure 3), showed a specific positive selection pressure in Palaeoheterodonta species. Indeed, the high Kr/Kc (>1.5) measured for each of the branches (Figure 3) suggest higher radical amino-acid substitution rates and thus more important modifications of biochemical properties of mitochondrial encoded proteins in Palaeoheterodonta. This lineage is characterized by freshwater species whereas Anomalodesmata, Heterodonta and Pteriomorphia are represented principally by marine species (Adamkewick et al., 1997). Freshwater and marine mollusks typically diverge in effective population sizes, with marine species usually having larger effective population sizes (Plowugh, 2016). This difference in effective population size could have played a role in the selective pressure experienced by mtPCGs and could provide elements of explanation, but it is not currently possible to determine the link between population sizes and energy metabolism. This would require further studies at the level of the individual gene coupled with biological analyses, to discriminate the genes and/or specific gene regions with high radical amino-acid substitution rate and to better understand the impact of these substitutions in the function of the encoded proteins.

### Relationship Among Life-History Traits

Considering only life-history traits, marginal correlations showed that longevity was highly positively correlated with generation time (*r* = 0.85; *pp* = 1) and negatively correlated with temperature (*r* = −0.47; *pp* = 0.012), and generation time was negatively correlated with temperature (*r* = −0.33; *pp* = 0.05) (Table 1). When the generation time was not considered in the model, the partial correlation between longevity and temperature was not as strong (*r* = −0.19; *pp* = 0.23) (Table 1). Similarly, generation time was no longer correlated with temperature (r = −0.03; *pp* = 0.46) when longevity was controlled (Table 1). This suggest that the strong relationship between longevity and generation time would be responsible for the marginal correlations observed for each one of these two traits with the mean temperature tolerance.

**TABLE 1:** Covariance analysis between life-history traits in 76 bivalve species.

Marginal correlations^a^	Generation time (years)			Mean temperature tolerance (°C)		
	*r*	*r* ^2^	*pp*	*r*	*r* ^2^	*pp*
Longevity (years)	0.8**	0.64	1	−0.47^**^	0.22	0.01
Generation time (years)	−	−	−	−0.33	0.11	0.05
**Partial correlations** ^b^	**Generation time (years)**			**Mean temperature tolerance (°C)**		
	***r***	***r*^*2*^**	***pp***	***r***	***r*^2^**	***pp***
Longevity (years)	0.85^**^	0.72	1	−0.19	0.036	0.23
Generation time (years)	−	−	−	−0.03	0.001	0.46

^a^ Correlation coefficients (r) corresponding to marginal correlations between each pair of variables.

^b^ Correlation coefficients (r) corresponding to partial correlations.

^*^ *PP* > 0.95 or <0.05.

^**^ *PP* > 0.975 or <0.025.

To determine the proportion of the variation observed in one trait that is predictable from the variation in another independent trait, we have also calculated the r-square (*r* ^2^) for each marginal and partial correlation. Therefore, the variation observed in longevity was predictable at 64% from the variation in generation time (*r* ^2^ = 0.64) and at 22% from the variation in mean temperature tolerance (Table 1), when considering the marginal correlations estimated between each pair of these three variables. For the partial correlations, 72 and 4% of the variation of longevity were predictable from the variation of generation time and temperature, respectively (Table 1), again reflecting the confounding effect of the strong correlation between longevity and generation in the results obtained for mean temperature tolerance through marginal correlations.

Bivalves with late maturation typically have longer lifespan than species with early maturation. Our results confirm the positive relationship between longevity and generation time found on a smaller subset of bivalves by Haag and Rypel (Haag and Rypel, 2011) and Ridgway et al. (Ridgway et al., 2011), and found also in birds and mammals (Lartillot and Pujol, 2011; Ridgway et al., 2011). Considerable efforts have been addressed to provide ultimate (evolutionary) explanations for the relationship between age at first reproduction and lifespan (Ljubuncic and Reznick, 2009) but mechanistic links are still missing. Temperature is known to have an important effect on both metabolic rate and ROS production (Samain, 2011; Blier et al., 2014) in ectotherms in general. It is therefore likely that these physiological characteristics associated with low temperature (for example, low metabolic rate and rare peak of ROS production in stable polar environments) help them live longer (Abele et al., 2009; Bodnar, 2009; Moss et al., 2016). Our analysis however clearly points out that age at maturation was not correlated with temperature, but instead the strong correlation of longevity with generation time explains the relationship observed in a marginal correlation. This therefore discards the hypothesis that temperature would be a major driver of early growth and maturation that would subsequently modulate lifespan. Our results suggest a closer and tighter mechanistic link between age at maturation and longevity independent of the impact of temperature on metabolism.

### Relationship Between mtPCGs Evolution and Life-History Traits

The synonymous substitution rate was negatively correlated with longevity (*r* = −0.3; *pp* = 0.04) and, to a lesser extent, with mean temperature tolerance (*r* = 0.31; *pp* = 0.92), but no significant marginal correlation was found with generation time (*r* = 0.09; *pp* = 0.69) (Table 2). The negative correlation between the substitution rate and longevity was stronger when the generation time and mean temperature tolerance were controlled (*r* = −0.57; *pp*=<0.01). Therefore in bivalves, based on the r-squared of marginal and partial correlations respectively, 9–30% of species longevity variation (marginal and partial correlations result, respectively) is associated with variation in mtDNA synonymous substitution rate. Surprisingly, we observed a strong positive correlation between dS and generation time (*r* = 0.58; *pp* = 1) when longevity and temperature were controlled (Table 2). In practice, the generation time increases with longevity. Thus, the positive relationship between generation time and dS could explain the less marked negative correlation between longevity and dS (*r* = 0.3; *pp* = 0.04) when confounding variables, such as generation time, are included in our model (marginal correlation) (Table 2). However, the negative relationship between longevity and dS is stronger and predominates over the positive relationship between generation time and dS because no correlation was found between generation time and dS (*r* = 0.09; *pp* = 0.69) when the variation of longevity is considered (marginal correlation) (Table 2).

**TABLE 2:** Covariance between dS, Kc, Kr/Kc and life-history traits in 76 bivalve species.

Marginal correlations^a^	Longevity (years)			Generation time (years)			Mean temperature tolerance (°C)		
	*r*	*r* ^*2*^	*pp*	*r*	*r* ^*2*^	*pp*	*r*	*r* ^*2*^	*pp*
dS	−0.3^*^	0.09	0.04	0.09	0.01	0.69	0.31	0.096	0.92
Kc	−0.28	0.08	0.07	-0.05	0.002	0.42	0.39	0.15	0.94
Kr/Kc	0.17	0.03	0.66	0.28	0.08	0.76	0.23	0.05	0.73
**Partial correlations^b^**	**Longevity (years)**			**Generation time (years)**			**Mean temperature tolerance (°C)**		
	***r***	***r*** ^*2*^	***pp***	***r***	***r*** ^*2*^	***pp***	***r***	***r*** ^*2*^	***pp***
dS	−0.57^**^	0.32	<0.01	0.58^**^	0.37	1	0.18	0.03	0.78
Kc	−0.28	0.08	0.14	0.3	0.09	0.86	0.27	0.073	0.82
Kr/Kc	0.06	0.004	0.58	0.3	0.09	0.8	0.45	0.2	0.88

^a^ Correlation coefficients (r) corresponding to the marginal correlations between each pair of variables.

^b^ Correlation coefficients (r) corresponding to the partial correlations.

^*^ *PP* > 0.95 or <0.05.

^**^ *PP* > 0.975 or <0.025.

These results, which suggest that long-lived bivalve species exhibit lower mtDNA substitution rates than short-lived species, are in agreement with the «mitochondrial theory of aging» (Nabholz et al., 2008; Galtier et al., 2009b). A similar negative relationship was also identified in mammals, birds and plants (Laroche et al., 1997; Laroche and Bousquet, 1999; Andreasen and Baldwin, 2001; Nabholz et al., 2008; Galtier et al., 2009a; Lartillot and Pujol, 2011; Lartillot and Delsuc, 2012; Lartillot, 2013). Thus, we provide here clear evidence that this correlation holds outside of vertebrates in animal kingdom. This study is also one of the first looking at the influence of temperature on mtDNA evolution and aging in ectotherms. Surprisingly, no relationship between dS and mean temperature tolerance was found. This might be due to the high level of missing values we observed. Studies on Archaea, protozoans, invertebrates, fishes, amphibians, reptiles, birds, mammals and plants all revealed that temperature explains a significant proportion of DNA mutations and substitution rates (Wright et al., 2003; Davies et al., 2004; Gillooly et al., 2005; Allen et al., 2006; Estabrook et al., 2007; Groussin and Gouy, 2011). Gillooly et al. (Gillooly et al., 2005) found that mtDNA evolution rate is higher for warmer-bodied endotherms than for ectothermic animals of similar size, attesting the potential impact of temperature on mtDNA evolution. Assuming that synonymous mutations are close to neutral, such that the synonymous substitution rate provides a good proxy for the mutation rate, the lack of impact of temperature on the rate of substitution in mtDNA entails low connection between metabolic rate and mutation rate. This partly invalidates the mechanistic “rate of living theory” (Pearl, 1928; Speakman et al., 2002), which advocates faster aging engendered by high metabolic rates. We could speculate that oxidative stress episodes, and mutation events, should correlate with intensity of energy metabolism since high proportion of ROS arises from mitochondrial respiration. Consequently, mutation rate should correlate with temperature considering higher metabolic rate in warmer environment, which is not what we obtained. Our results are at odds with those obtained on poison frogs (Santos, 2012) for which the rate of evolution of nuclear and mitochondrial DNA are correlated with active metabolic rate (AMR) but not with standard metabolic rate (SMR). As mentioned in Santos (2012), the relationship between AMR and rate of evolution could be explained by the association with lifespan. Unfortunately, reliable data on lifespan were missing to evaluate this longevity hypothesis. The suspected direct and strong link between metabolic rate and oxidative stress implies the low capacity of organism to manage oxidative stress. Studying the longest-lived bivalve *Arctica islandica*, Munro et al. (Munro et al., 2013) have shown that mitochondria of this species generate much less hydrogen peroxide at a given respiration rate than two shorter-lived species. They therefore suggested that delayed aging process may be the consequence of the evolution of mitochondrial function, which minimize oxidative stress in physiological conditions independently of occurrence and intensity of mitochondrial activity. It is then conceivable that divergences in optimal or upper lethal temperatures will have low impact on both oxidative stress and mutation rate. While mitochondrial oxidative stress theory has received some support (Hulbert et al., 2007; Blier et al., 2017), the direct role of oxidative stress in explaining species divergences in the rate of aging is still debated. One of the challenges is to link proximal (mechanistic) theory to ultimate (evolutionary) theory of aging and accordingly, in the case of mitochondrial theory of oxidative stress establish a connection between management of oxidative stress and age at reproduction. One potential avenue to address this question is to explore cross-communication between ROS management (or Nitrogen Reactive Species) and regulation pathways that coordinate bioenergetics, early growth, and sexual maturation: the insuline/insulin-like growth factor 1 (IGF-1), the mechanistic target of rapamycin (mTOR), and sirtuins (Narasimhan et al., 2009; Rollo, 2010) or their homologs. Since in this wide taxonomic comparison, age at reproduction is clearly associated to longevity, bivalves should be a good comparative model to resolve this conundrum.

A previous study in mammals has shown positive correlation of dN/dS ratios with longevity in mammals (Lartillot and Pujol, 2011), which was interpreted in the light of the nearly-neutral theory, as an indirect consequence of variation in effective population size between short- and long-lived species. Our model failed to detect a relationship of dN/dS with any of the life-history traits in bivalves. We cannot discard the possibility that this lack of correlation could be explained by high level of saturation of mt-DNA substitutions related to long evolutionary history of studied species (the node of our phylogenetic trees being over 500 My distant). Ultimately, correlation analyses between dN/dS and more direct proxies of effective population size (e.g., nucleotide diversity, see Figuet et al. (2014)) could be conducted.

We used also the Kr/Kc ratio to evaluate the fixation rate of either slightly deleterious or adaptive mutations in mtDNA (Zhang, 2000; Smith, 2003; Hanada et al., 2007; Popadin et al., 2007; Nabholz et al., 2013), as saturation levels of amino acid substitutions may be lower than the nucleotide one and Kr/Kc shows a stronger relationship with life-history traits than dN/dS (Nabholz et al., 2013). Galtier et al. (Galtier et al., 2009b) reported a negative relationship between Kc and longevity in birds and mammals. Likewise, in mammals, the Kr/Kc ratio correlates positively with body mass or age at sexual maturity (Nikolaev et al., 2007; Popadin et al., 2007). Here, no significant correlation was detected between Kr/Kc and any of the three life-history traits (Table 2). The correlations between conservative amino acid replacement rate (Kc) and the three life-history traits (longevity, maturity and mean temperature tolerance) were weak and non-significant based on marginal and partial correlations (Table 2). Only longevity and mean temperature tolerance seem to be respectively negatively (*r* = −0.28; *pp* = 0.07) and positively (*r* = 0.39; *pp* = 0.94) correlated with Kc through marginal correlations (Table 2).

Again, long evolutionary history and high level of mutations accumulations may have resulted in a hardly detectable evolutionary signal (Plazzi and Passamonti, 2010). Alternatively, the negative correlation between population size and longevity, which was invoked to explain the correlation patterns of dN/dS and Kr/Kc in birds and mammals, may not hold in mollusks. Further studies are required to carefully explore this relation between life-history traits and mutation rate. Moreover, we have not considered the impact of other parameters such as the air exposure, which could have an impact on the mitochondrial energy production and thus could affect the longevity and the age at sexual maturity in bivalves. It may also be interesting to investigate about this relationship by focusing on specific bivalve subgroups of species, such as the slow-evolving Palaeoheterodonta species, or subgroup of species which have been adapted to unusual “extreme” environment (as hydrothermal vent bivalves or Arctic and Antarctic species). Finally, we did not explore the impact of compositional variation between lineages on the overall correlation analysis, another aspect that might require further investigation.

## Conclusion

Here, we performed a first preliminary analysis of evolutionary signals in mitochondrial DNA potentially linked to life-history traits in bivalves. Our results confirm the known strong positive correlation between longevity and generation time (Ljubuncic and Reznick, 2009), and clearly establish a negative relationship between substitution rates and longevity in a group of invertebrates expanding this correlation previously documented for vertebrates (Laroche et al., 1997; Laroche and Bousquet, 1999; Andreasen and Baldwin, 2001; Nabholz et al., 2008; Galtier et al., 2009a; Lartillot and Pujol, 2011; Lartillot and Delsuc, 2012; Lartillot, 2013). This correlation is not mediated by either temperature or age at maturity. In the future, it will be of interest to identify which of the different mtPCGs are involved and by focusing on specific group of species, like the Palaeheterodonta species, which present unusual characteristics in their mtDNA evolution.

## Materials and Methods

### Molecular and Life History Trait Data

Sixty five complete female mitochondrial genomes were downloaded from GenBank (Benson et al., 2013). Eleven mitochondrial genomes sequenced by our group were added to the dataset (Leviviers et al. in prep). In all, the mitochondrial genomes of 76 bivalve species were used for our analyses (Supplementary Table S1). Three life-history traits were tested: longevity, generation time, and mean temperature tolerance. We used the published records of maximum life span as a proxy for longevity, the age of female at sexual maturity as a proxy for generation time, and the maximum lethal temperature measured in laboratory as a proxy for maximum lethal temperature in environment (37 datas were found for both longevity and generation time and 24 were found for maximum lethal temperature, Supplementary Tables S1–S7).

### Phylogenetic Reconstruction

A phylogenetic tree was then inferred with the PhyloBayes program (Lartillot and Philippe, 2004) using a CAT GTR GAMMA four model with two Monte Carlo Markov chains running simultaneously. As ATP8 is often absent or highly modified within bivalves we did not consider this gene for the phylogenetic analyses (Gissi et al., 2008). Each 12 protein coding gene sequences (PCG) from the 76 bivalve mtDNAs were first aligned separately using MUSCLEv3.8.31 (Edgar, 2004) and the poorly aligned positions were removed using Gblocks v.0.91b (Catresana, 2000) with default parameters. Then the PCG were concatenated resulting in 1,645 amino acids. A Bayesian tree was obtained under the site-heterogeneous CAT-GTR model using PhyloBayes 3.3f (Lartillot et al., 2009). The CAT-GTR model was used because of its increased robustness against long-branch attraction artifacts in the presence of mutational saturation (Lartillot et al., 2007; Lartillot et al., 2013). Two independent Monte Carlo Markov chains were run, for 5,000 cycles (each comprising a large number of generations, cycling over updates of all components of the parameter vector), excluding the first 400 cycles (burn-in). The two consensus trees were nearly identical between the two independent runs (maximum difference between posterior probability support of 0.08 over all clades). The protobranchs, *Solemya velum* (accession: NC_017612) and *Nucula nucleus* (accession: EF211991) were used as outgroup for phylogenetic analyses. Overall mean p-distance were measured for the three groups of Heterodonta, Pteriomorphia and Palaeheterodonta species through the MEGA07 software (Kumar et al., 2016).

### Relationship Among Life History Traits

The correlation and covariation among life history traits and mtDNA evolution were estimated using a Bayesian framework with a Markov chain Monte Carlo (MCMC) sampling approach as implemented in CoEvol 1.4b (Lartillot and Pujol, 2011) (http://github.com/bayesiancook/coevol.git). The strength of the correlation between the evolution rate and life-history traits is given as a posterior probability (*pp*) of a positive (*pp* close to 1) or a negative correlation (*pp* close to 0). The mt-DNA evolution was represented by considering the synonymous substitution rate (dS), the ratio of nonsynonymous over synonymous substitution rates (dN/dS), the ratio of a radical over conservative amino acid replacement rates (Kr/Kc) and the conservative amino-acid replacement rate (Kc). Under the Kr/Kc model, substitutions were considered as radical if they change the polarity or the volume of the amino-acid. Two independent runs were performed for each analysis, during 3,200 cycles excluding the first 200 cycles (burn-in) for dS analysis, and 5,000 cycles excluding the first 200 cycles for Kr/Kc analysis. Their convergence was assessed by measuring several key statistics (log likelihood, mean substitution rate over the tree, mean omega over the tree, entries of the covariance matrix, root age, etc.), the effective sample size, and the discrepancy between the credibility intervals obtained from the two independent runs. For all these statistics, the effective sample size was greater than 500 and the relative discrepancy was less than 0.12.

## Data Availability

The datasets presented in this study can be found in online repositories. The names of the repository/repositories and accession number(s) can be found in the article/Supplementary Material.

## References

[B1] Abele D. Brey T. Philipp E. (2009). Bivalve models of aging and the determination of molluscan lifespans. Exp. Gerontol. 44, 307–315. 10.1016/j.exger.2009.02.012 19268513

[B2] Abele D. Philipp E. (2013). Environmental control and control of the environment: the basis of longevity in bivalves. Gerontology 59, 261–266. 10.1159/000345331 23257622

[B3] Adamkewicz S. L. Harasewych M. G. Blake J. Saudek D. Bult C. J. (1997). A molecular phylogeny of the bivalve mollusks. Mol. Biol. Evol. 14, 619–629. 10.1371/journal.pone.0027147 9190063

[B4] Allen A. P. Gillooly J. F. Savage V. M. Brown J. H. (2006). Kinetic effects of temperature on rates of genetic divergence and speciation. PNAS 103, 9130–9135. 10.1073/pnas.0603587103 16754845 PMC1474011

[B5] Andreasen K. Baldwin B. G. (2001). Unequal evolutionary rates between annual and perennial lineages of checker mallows (*Sidalcea*, Malvaceae): evidence from 18S–26S rDNA internal and external transcribed spacers. Mol. Biol. Evol. 18, 936–944. 10.1093/oxfordjournals.molbev.a003894 11371581

[B6] Barja G. (2004). Free radicals and aging. Trends Neurosci. 27, 595–600. 10.1016/j.tins.2004.07.005 15374670

[B7] Bauer G. (1987). Reproductive strategy of the freshwater pearl mussel *Margaritifera margaritifera* . J. Anim. Ecol. 56, 691–704. 10.2307/5077

[B8] Benson D. A. Karsch-Mizrachi I. Lipman D.J. Ostell J. Sayers E. W. (2013). GenBank. Nucleic Acids Res. 41, 36–42. 10.1093/nar/gkn723 PMC3531190 23193287

[B9] Bieler R. Mikkelsen P. M. Collins T. M. Glover E. A. González V. L. Graf D. L. (2014). Investigating the Bivalve tree of life–an exemplar-based approach combining molecular and novel morphological characters. Inverteb. Syst. 28 (1), 32. 10.1071/IS13010

[B10] Blier P. U. Abele D. Munro D. Degletagne C. Rodriguez E. Hagen T. (2017). What modulates animal longevity? Fast and slow aging in bivalves as a model for the study of lifespan. Semin. Cell Dev. Biol. 70, 130. 10.1016/j.semcdb.2017.07.046 28778411

[B11] Blier P. U. Lemieux H. Pichaud N. (2014). Holding our breath in our modern world: will mitochondria keep the pace with climate changes ?. Can. J. Zool. 92, 591–601. 10.1139/cjz-2013-0183

[B12] Bodnar A. G. (2009). Marine invertebrates as models for aging research. Exp. Gerontol. 44, 477–484. 10.1016/j.exger.2009.05.001 19454313

[B13] Breton S. Burger G. Stewart D. T. Blier P. U. (2006). Comparative analysis of gender-associated complete mitochondrial genomes in marine mussels (Mytilus spp.). Genetics 172, 1107–1119. 10.1534/genetics.105.047159 16322521 PMC1456209

[B14] Bureau D. (2002). Age, size structure and growth parameters of geoducks (*Panopea abrupta*, Conrad 1849) from 34 locations in British Columbia sampled between 1993 and 2000. Can. Tech. Rep. Fish. Aquat. Sci. 2413, 29.

[B15] Butler P. G. Wanamaker A. D. Scourse J. D. Richardson C. A. Reynolds D. J. (2012). Variability of marine climate on the North Icelandic Shelf in a 1357-year proxy archive based on growth increments in the bivalve *Arctica islandica* . Palaeogeogr. Palaeoclimatol. Palaeoecol. 373, 141–151. 10.1016/j.palaeo.2012.01.016

[B16] Cardoso R. Veloso V. (2003). Population dynamics and secondary production of the wedge clam *Donax hanleyanus* (Bivalvia : Donacidae) on a high-energy, subtropical beach of Brazil. Mar. Biol. 142, 153–162. 10.1007/s00227-002-0926-2

[B17] Castresana J. (2000). Selection of conserved blocks from multiple alignments for their use in phylogenetic analysis. Mol. Biol. Evol. 17, 540–552. 10.1093/oxfordjournals.molbev.a026334 10742046

[B18] Cope J. (1996). Origin and evolutionary radiation of the Mollusca. Editor Taylor J. (New York, NY: Oxford University Press), 361–370.

[B19] Currais A. (2015). Aging and inflammation–a central role for mitochondria in brain health and disease. Aging Res. Rev. 21, 30–42. 10.1016/j.arr.2015.02.001 25684584

[B20] Davies T. J. Savolainen V. Chase M. W. Moat J. Barraclough T. G. (2004). Environmental energy and evolutionary rates in flowering plants. Proc. R. Soc. B 271, 2195–2200. 10.1098/rspb.2004.2849 PMC1691837 15475341

[B21] Dégletagne C. Abele D. Held C. (2016). A distinct mitochondrial genome with DUI-like inheritance in the Ocean Quahog *Arctica islandica* . Mol. Biol. Evol. 33 (2), 375–383. 10.1093/molbev/msv224 26486872 PMC4866540

[B22] Doucet Beaupré H. Breton S. Chapman G. E. Blier P. U. Bogan A. E. Stewart D. T. Hoeh W. R. (2010). Mitochondrial phylogenomics of the Bivalvia (Mollusca): searching for the origin and mitogenomic correlates of doubly uniparental inheritance of mt-DNA. BMC Evol. Biol. 10, 50. 10.1186/1471-2148-10-50 20167078 PMC2834691

[B23] Dreyer H. Steiner G. Harper E. M. (2003). Molecular phylogeny of Anomalodesmata (Mollusca: Bivalvia) inferred from 18S rRNA sequences. Zool. J. Linn. Soc. 139, 229–246. 10.1046/j.1096-3642.2003.00065.x

[B24] Edgar R. C. (2004). MUSCLE: multiple sequence alignment with high accuracy and high throughput. Nucleic Acids Res. 32, 1792–1797. 10.1093/nar/gkh340 15034147 PMC390337

[B25] Estabrook G. F. Smith G. R. Dowling T. E. (2007). Body mass and temperature influence rates of mitochondrial DNA evolution in North American cyprinid fish. Evolution 61, 1176–1187. 10.1111/j.1558-5646.2007.00089.x 17492970

[B26] Feng J. Bussière F. Hekimi S. (2001). Mitochondrial electron transport is a key determinant of life span in *Caenorhabditis elegans* . Dev. Cell 1, 633–644. 10.1016/s1534-5807(01)00071-5 11709184

[B27] Figuet E. Romiguier J. Dutheil J. Y. Galtier N. (2014). Mitochondrial DNA as a tool for reconstructing past life-history traits in mammals. J. Evol. Biol. 27, 899–910. 10.1111/jeb.12361 24720883

[B28] Galtier N. Blier P. U. Nabholz B. (2009a). Inverse relationship between longevity and evolutionary rate of mitochondrial proteins in mammals and birds. Mitochondrion 9, 51–57. 10.1016/j.mito.2008.11.006 19154799

[B29] Galtier N. Jobson R. W. Nabholz B. Glémin S. Blier P. U. (2009b). Mitochondrial whims: metabolic rate, longevity and the rate of molecular evolution. Biol. Lett. 5, 413–416. 10.1098/rsbl.2008.0662 19324654 PMC2679905

[B30] Gillooly J. F. Allen A. P. West G. B. Brown J. H. (2005). The rate of DNA evolution: effects of body size and temperature on the molecular clock. PNAS 102, 140–145. 10.1073/pnas.0407735101 15618408 PMC544068

[B31] Giribet G. Wheeler W. (2002). On bivalve phylogeny: a high-level analysis of the Bivalvia (Mollusca) based on combined morphology and DNA sequence data. Invertebr. Biol. 121, 271–324. 10.1111/j.1744-7410.2002.tb00132.x

[B32] Gissi C. Iannelli F. Pesole G. (2008). Evolution of the mitochondrial genome of Metazoa as exemplified by comparison of congeneric species. Heredity 101, 301–320. 10.1038/hdy.2008.62 18612321

[B93] González V. L. Andrade S. C. Bieler R. Collins T. M. Dunn C. W. Mikkelsen P. M. (2015). A phylogenetic backbone for Bivalvia: an RNA-seq approach. Proc. Biol. Sci. 282 (1801), 20142332. 25589608 10.1098/rspb.2014.2332 PMC4308999

[B33] Groussin M. Gouy M. (2011). Adaptation to environmental temperature is a major determinant of molecular evolutionary rates in Archaea. Mol. Biol. Evol. 28, 2661–2674. 10.1093/molbev/msr098 21498602

[B34] Guo X. (2009). Use and exchange of genetic resources in molluscan aquaculture. Rev. Aquac. 1, 251–259. 10.1111/j.1753-5131.2009.01014.x

[B35] Haag W. R. Rypel A. L. (2011). Growth and longevity in freshwater mussels: evolutionary and conservation implications. Biol. Rev. 86, 225–247. 10.1111/j.1469-185X.2010.00146.x 20608928

[B36] Hanada K. Shiu S. H. Li W. H. (2007). The nonsynonymous/synonymous substitution rate ratio versus the radical/conservative replacement rate ratio in the evolution of mammalian genes. Mol. Biol. Evol. 24, 2235–2241. 10.1093/molbev/msm152 17652332

[B37] Harman D. (1956). Aging: a theory based on free radical and radiation chemistry. J. Gerontol. 11, 298–300. 10.1093/geronj/11.3.298 13332224

[B38] Hua X. Cowman P. Warren D. Bromham L. (2015). Longevity is linked to mitochondrial mutation rates in rockfish: a test using Poisson regression. Mol. Biol. Evol. 32, 2633–2645. 10.1093/molbev/msv137 26048547

[B39] Hughes K. A. Reynolds R. M. (2005). Evolutionary and mechanistic theories of aging. Annu. Rev. Entomol. 50, 421–445. 10.1146/annurev.ento.50.071803.130409 15355246

[B40] Hulbert A. J. Pamplona R. Buffenstein R. Buttemer W. A. (2007). Life and death: metabolic rate, membrane composition, and life span of animals. Physiol. Rev. 87, 1175–1213. 10.1152/physrev.00047.2006 17928583

[B41] Kirkwood T. (1977). Evolution of aging. Nature 270, 301–304. 10.1038/270301a0 593350

[B42] Kirkwood T. Holliday R. (1979). The evolution of aging and longevity. Proc. R. Soc. B 205, 531–546. 10.1098/rspb.1979.0083 42059

[B43] Kumar S. Stecher G. Tamura K. Dudley J. (2016). MEGA7: molecular evolutionary genetics analysis version 7.0 for bigger datasets downloaded from. Mol. Biol. Evol. 33 (7), 1870–1874. 10.1093/molbev/msw054 27004904 PMC8210823

[B44] Laroche J. Bousquet J. (1999). Evolution of the mitochondrial rps3 intron in perennial and annual angiosperms and homology to nad5 intron 1. Mol. Biol. Evol. 16, 441–452. 10.1093/oxfordjournals.molbev.a026126 10331271

[B45] Laroche J. Li P. Maggia L. Bousquet J. (1997). Molecular evolution of angiosperm mitochondrial introns and exons. Proc. Natl. Acad. Sci. U.S.A. 94, 5722–5727. 10.1073/pnas.94.11.5722 9159140 PMC20846

[B46] Lartillot N. Brinkmann H. Philippe H. (2007). Suppression of long-branch attraction artefacts in the animal phylogeny using a site-heterogeneous model. BMC Evol. Biol. 7, (Suppl. 1), S4. 10.1186/1471-2148-7-S1-S4 PMC1796613 17288577

[B47] Lartillot N. Delsuc F. (2012). Joint reconstruction of divergence times and life-history evolution in placental mammals using phylogenetic covariance model. Evolution 66, 1773–1787. 10.1111/j.1558-5646.2011.01558.x 22671546

[B48] Lartillot N. (2013). Interaction between selection and biased gene conversion in mammalian protein-coding sequence evolution revealed by a phylogenetic covariance analysis. Mol. Biol. Evol. 30, 356–368. 10.1093/molbev/mss231 23024185

[B49] Lartillot N. Lepage T. Blanquart S. (2009). PhyloBayes 3: a Bayesian software package for phylogenetic reconstruction and molecular dating. Bioinformatics 25, 2286–2288. 10.1093/bioinformatics/btp368 19535536

[B50] Lartillot N. Philippe H. (2004). A Bayesian mixture model for across-site heterogeneities in the amino-acid replacement process. Mol. Biol. Evol. 21, 1095–1109. 10.1093/molbev/msh112 15014145

[B51] Lartillot N. Poujol R. (2011). A phylogenetic model for investigating correlated evolution of substitution rates and continuous phenotypic characters. Mol. Biol. Evol. 28, 729–744. 10.1093/molbev/msq244 20926596

[B52] Lartillot N. Rodrigue N. Stubbs D. Richer J. (2013). PhyloBayes MPI: phylogenetic reconstruction with infinite mixtures of profiles in a parallel environment. Syst. Biol. 62 (4), 611–615. 10.1093/sysbio/syt022 23564032

[B53] Li X.-X. Tsoi B. Li Y.-F. Kurihara H. He R.-R. (2015). Cardiolipin and its different properties in mitophagy and apoptosis. J. Histochem. Cytochem. 63, 301–311. 10.1369/0022155415574818 25673287 PMC4409943

[B54] Ljubuncic P. Reznick A. Z. (2009). The evolutionary theories of aging revisited - a mini-review. Gerontology 55, 205–216. 10.1159/000200772 19202326

[B55] Medawar P. (1952). An unsolved problem of biology. London, United Kingdom: H.K. Lewis and Co., 24.

[B56] Mistri M. (2002). Ecological characteristics of the invasive Asian date mussel, *Musculista senhousia*, in the Sacca Di Goro (Adriatic Sea, Italy). Estuaries 25, 431–440. 10.1007/BF02695985

[B57] Moss D. K. Ivany L. C. Judd E. J. Cummings P. W. Bearden C. E. Kim W. J. (2016). Life span, growth rate, and body size across latitude in marine Bivalvia, with implications for Phanerozoic evolution. Proc. R. Soc. B. 283, 1364. 10.1098/rspb.2016.1364 PMC5013775 27488653

[B58] Munro D. Blier P. U. (2012). The extreme longevity of *Arctica islandica* is associated with increased peroxidation resistance in mitochondrial membranes. Aging Cell 11, 845–855. 10.1111/j.1474-9726.2012.00847.x 22708840

[B59] Munro D. Pichaud N. Paquin F. Kemeid V. Blier P. U. (2013). Low hydrogen peroxide production in mitochondria of the long-lived *Arctica islandica*: underlying mechanisms for slow aging. Aging Cell 12, 584–592. 10.1111/acel.12082 23566066

[B60] Nabholz B. Glémin S. Galtier N. (2008). Strong variations of mitochondrial mutation rate across mammals–the longevity hypothesis. Mol. Biol. Evol. 25, 120–130. 10.1093/molbev/msm248 17998254

[B61] Nabholz B. Künstner A. Wang R. Jarvis E. D. Ellegren H. (2011). Dynamic evolution of base composition: causes and consequences in avian phylogenomics. Mol. Biol. Evol. 28, 2197–2210. 10.1093/molbev/msr047 21393604 PMC3144382

[B62] Nabholz B. Uwimana N. Lartillot N. (2013). Reconstructing the phylogenetic history of long-term effective population size and life-history traits using patterns of amino acid replacement in mitochondrial genomes of mammals and birds. Genome Biol. Evol. 5, 1273–1290. 10.1093/gbe/evt083 23711670 PMC3730341

[B63] Narasimhan S. D. Yen K. Tissenbaum H. A. (2009). Converging pathways in lifespan regulation. Curr. Biol. 19, R657–R666. 10.1016/j.cub.2009.06.013 19674551 PMC3109866

[B64] Nicholls D. G. Ferguson S. J. (2013). Bioenergetics. 4th Edn. Amsterdam: Academic Press, 343.

[B94] Nikolaev S. L. Montoya-Burgos J. I. Popadin K. Parand L. Margulies E. H. Antonarakis S. E., & National Institutes of Health Intramural Sequencing Center Comparative Sequencing Program (2007). Life-history traits drive the evolutionary rates of mammalian coding and noncoding genomic elements. PNAS 104 (51), 20443–20448. 18077382 10.1073/pnas.0705658104 PMC2154450

[B65] Pamplona R. Barja G. (2011). An evolutionary comparative scan for longevity-related oxidative stress resistance mechanisms in homeotherms. Biogerontology 12, 409–435. 10.1007/s10522-011-9348-1 21755337

[B66] Pearl R. (1928). The rate of living, Being an Account of Some Experimental Studies on the Biology of Life Duration. New York, NY: Alfred A. Knopf, 185.

[B67] Philipp E. Pörtner H.-O. Abele D. (2005). Mitochondrial aging of a polar and a temperate mud clam. Mech. Aging Dev. 126, 610–619. 10.1016/j.mad.2005.02.002 15811430

[B68] Plazzi F. Ceregato A. Taviani M. Passamonti M. (2011). A molecular phylogeny of bivalve mollusks: Ancient radiations and divergences as revealed by mitochondrial genes. PLoS One 6, 1–16. 10.1371/journal.pone.0027147 PMC3206082 22069499

[B69] Plazzi F. Passamonti M. (2010). Towards a molecular phylogeny of Mollusks: Bivalves’ early evolution as revealed by mitochondrial genes. Mol. Phylogenet. Evol. 57, 641–657. 10.1016/j.ympev.2010.08.032 20817110

[B70] Plough L. V. (2016). Genetic load in marine animals: a review. Curr. Zoolog. 62 (6), 567–579. 10.1093/cz/zow096 PMC5804265 29491946

[B71] Popadin K. Polishchuk L. V. Mamirova L. Knorre D. Gunbin K. (2007). Accumulation of slightly deleterious mutations in mitochondrial protein-coding genes of large versus small mammals. Proc. Natl. Acad. Sci. U.S.A. 104, 13390–13395. 10.1073/pnas.0701256104 17679693 PMC1948928

[B72] Rath E. Haller D. (2012). Mitochondria at the interface between danger signaling and metabolism: role of unfolded protein responses in chronic inflammation. Inflamm. Bowel Dis. 18, 1364–1377. 10.1002/ibd.21944 22183876

[B73] Ren M. Phoon C. K. L. Schlame M. (2014). Metabolism and function of mitochondrial cardiolipin. Prog. Lipid Res. 55, 1–16. 10.1016/j.plipres.2014.04.001 24769127

[B74] Richardson C. (2001). Molluscs as archives of environmental change. Oceanogr. Mar. Biol. 39, 103–164.

[B75] Ridgway I. D. Richardson C. A. Austad S. N. (2011). Maximum shell size, growth rate, and maturation age correlate with longevity in bivalve molluscs. Journals Gerontol 66A, 183–190. 10.1093/gerona/glq172 PMC3107019 20966102

[B76] Rollo C. D. (2010). Aging and the mammalian regulatory triumvirate. Aging Dis. 1, 105–138. 22396860 PMC3295031

[B77] Samain J.-F. (2011). Review and perspectives of physiological mechanisms underlying genetically-based resistance of the Pacific oyster *Crassostrea gigas* to summer mortality. Aquat. Living Resour. 24, 227–236. 10.1051/alr/2011144

[B78] \Santos J. C. (2012). Fast molecular evolution associated with high active metabolic rates in poison frogs. Mol. Biol. Evol. 29, 2001–2018. 10.1093/molbev/mss069 22337863

[B79] Silva-Cavalcanti J. S. Costa M. F. (2011). Fisheries of *Anomalocardia brasiliana* in tropical estuaries. Panam. J. Aquat. Sci. 6, 86–99.

[B80] Smith D. R. Snyder M. (2007). Complete mitochondrial DNA sequence of the scallop *Placopecten magellanicus*: evidence of transposition leading to an uncharacteristically large mitochondrial genome. J. Mol. Evol. 65, 380–391. 10.1007/s00239-007-9016-x 17922075

[B81] Smith N. G. C. (2003). Are radical and conservative substitution rates useful statistics in molecular evolution?. J. Mol. Evol. 57, 467–478. 10.1007/s00239-003-2500-z 14708579

[B82] Speakman J. R. Selman C. McLaren J. S. Harper E. J. (2002). Living fast, dying when? The link between aging and energetics. J. Nutr. 132, 1583S–1597S. 10.1093/jn/132.6.1583S 12042467

[B83] Tacutu R. Craig T. Budovsky A. Wuttke D. Lehmann G. Taranukha D. Costa J. (2013). Human aging genomic resources: integrated databases and tools for the biology and genetics of aging. Nucleic Acids Res. 41, 1027–1033. 10.1093/nar/gks1155 PMC3531213 23193293

[B84] Thoudam T. Jeon J. H. Ha C. M. Lee I. K. (2016). Role of mitochondria-associated endoplasmic reticulum membrane in inflammation-mediated metabolic diseases. Mediators Inflamm. 2016, 1–18. 10.1155/2016/1851420 PMC5198184 28074080

[B85] Trifunovic A. Wredenberg A. Falkenberg M. Spelbrink J. N. Rovio A. T. Bruder C. E. Bohlooly-Y M. (2004). Premature aging in mice expressing defective mitochondrial DNA polymerase. Nature 429, 417–423. 10.1038/nature02517 15164064

[B86] Williams G. (1957). Pleiotropy, natural selection, and the evolution of senescence. Evolution 11, 398–411. 10.2307/2406060

[B87] Wright S. D. Gray R. D. Gardner R. C. (2003). Energy and the rate of evolution: inferences from plant rDNA substitution rates in the western pacitic. Evolution 57, 2893–2898. 10.1111/j.0014-3820.2003.tb01529.x 14761066

[B88] Xu W. Jameson D. Tang B. Higgs P. G. (2006). The relationship between the rate of molecular evolution and the rate of genome rearrangement in animal mitochondrial genomes. J. Mol. Evol. 63, 375–392. 10.1007/s00239-005-0246-5 16838214

[B89] Yang J. N. Seluanov A. Gorbunova V. (2013). Mitochondrial inverted repeats strongly correlate with lifespan: mt-DNA inversions and aging. PLoS One 8, e73318. 10.1371/journal.pone.0073318 24069185 PMC3775743

[B90] Zhang J. (2000). Rates of conservative and radical nonsynonymous nucleotide substitutions in mammalian nuclear genes. J. Mol. Evol. 50, 56–68. 10.1007/s002399910007 10654260

[B91] Ziuganov V. Miguel E. S. Neves R. J. Longa A. Fernández C. Amaro R. (2000). Life span variation of the freshwater pearl shell: a model species for testing longevity mechanisms in animals. AMBIO 29, 102–105. 10.1579/0044-7447-29.2.102

[B92] Zouros E. (2013). Biparental inheritance through uniparental transmission: the doubly uniparental inheritance (DUI) of mitochondrial DNA. Evol. Biol., 40 (1), 1–31. 10.1007/s11692-012-9195-2

